# Cardiovascular outcomes in adults with migraine treated with eptinezumab for migraine prevention: pooled data from four randomized, double-blind, placebo-controlled studies

**DOI:** 10.1186/s10194-021-01360-1

**Published:** 2021-11-25

**Authors:** Timothy R. Smith, Egilius L. H. Spierings, Roger Cady, Joe Hirman, Anders Ettrup, Vivienne Shen

**Affiliations:** 1 StudyMetrix Research, 3862 Mexico Road, St. Peters, MO 63303 USA; 2 Medvadis Research Corporation, Boston PainCare, Waltham, MA USA; 3 Lundbeck LLC, Deerfield, IL USA; 4 Pacific Northwest Statistical Consulting, Inc., Woodinville, WA USA; 5 H. Lundbeck A/S, Copenhagen, Denmark

**Keywords:** Eptinezumab, Cardiovascular, CGRP, Migraine

## Abstract

**Background:**

Patients with migraine have an increased relative risk of cardio- and cerebrovascular events, and some migraine treatments may exacerbate this risk. The primary objective of this analysis was to determine whether the rate of cardiovascular adverse events was higher for patients with migraine treated with the migraine-preventive eptinezumab, compared with patients receiving placebo.

**Methods:**

Cardiovascular outcomes in patients with migraine were pooled across four clinical trials (phase 1b, phase 2, and two phase 3 trials) for use of eptinezumab as a preventive migraine treatment for up to 1 year. In all studies, treatment-emergent adverse events (TEAEs) that occurred after the first dose of study treatment (eptinezumab 100 mg, 300 mg, 1000 mg, or placebo) and vital signs were recorded through study completion.

**Results:**

Cardiovascular TEAEs were rare across all four clinical trials, and rates were similar between patients receiving eptinezumab and those receiving placebo. Cardiovascular TEAEs that did occur were mild or moderate in severity; there were no serious adverse events as per FDA definition. Vital signs (systolic blood pressure, diastolic blood pressure, and heart rate) were not meaningfully different across treatment groups over the course of 56 weeks, compared to placebo. Treatment with eptinezumab did not result in significant new or changed cardiovascular medications used concomitantly compared to placebo.

**Conclusions:**

In this post hoc analysis of four clinical trials for eptinezumab, doses of 100 mg, 300 mg, and 1000 mg (more than 3 times the highest approved dose) were not associated with clinically relevant changes in vital signs or significant changes in concomitant cardiovascular medication usage, and had low incidences of cardiovascular TEAEs, comparable to placebo.

**Trial registration:**

NCT01772524 (Study 2), 01/21/2013; NCT02275117 (Study 5), 10/27/2014; NCT02559895 (PROMISE-1), 09/25/2017; NCT02974153 (PROMISE-2), 11/28/2016

**Supplementary Information:**

The online version contains supplementary material available at 10.1186/s10194-021-01360-1.

## Background

Affecting more than 1 billion people worldwide, migraine is a major cause of disability, especially in young adult and middle-aged women [1, 2]. Patients suffering from migraine, especially those experiencing migraine with aura, have an increased relative risk for vascular comorbidities and events, such as stroke and myocardial ischemia, as compared to patients without migraine (or migraine without aura) [3–7]. Given the high frequency of both migraine and cardiovascular risk factors in the general population, it is important to assess the coexistence of these two risk factors and the potential impact of long-term migraine treatment.

Calcitonin gene-related peptide (CGRP) is currently thought to be central in migraine headache pathophysiology [8, 9] and is known to be a microvascular vasodilator [10]. There are currently four monoclonal antibodies approved by the U.S. Food and Drug Administration (FDA) for the prevention of migraine that either bind to the CGRP ligand (fremanezumab-vfrm; galcanezumab-glnm; and eptinezumab-jjmr) or to the canonical CGRP receptor (erenumab-aooe [11]). Three have also been approved by the European Medicines Agency (fremanezumab-vfrm; galcanezumab-glnm; and erenumab-aooe). In addition, small-molecule CGRP receptor antagonists are approved by the FDA for acute treatment of migraine (rimegepant [12] and ubrogepant [13]) and migraine prevention (rimegepant [12] and atogepant [14]). Because CGRP mediates vasodilation [10], any therapy that inhibits the CGRP pathway may antagonize compensatory vasodilation. A study in isolated rat hearts found that CGRP is able to attenuate myocardial ischemia induced by a vasoconstrictor peptide [15]. Recently, a study in mice found that gepants (olcegepant and rimegepant), which are CGRP-receptor antagonists, worsened ischemic stroke [16], raising concerns that CGRP antagonism may increase the potential risk in patients at-risk for stroke or myocardial infarction. However, the overall importance of the CGRP pathway, when compared with other vasodilatory pathways, during ischemia (e.g., myocardial ischemia) is yet to be established [6, 10].

Acute migraine-specific medications (such as triptans and ergots) may rarely cause clinically meaningful vasoconstriction [17], and evidence of significant cardiovascular risk with triptans is lacking. Post-marketing data for erenumab, a CGRP-receptor antibody indicated for the preventive treatment of migraine in adults, suggest development or worsening of hypertension, leading to inclusion under Warnings and Precautions in its prescribing information [11]. However, results from a long-term, open-label extension study found erenumab to be safe in populations at cardiovascular risk [18]. Post-market surveillance of CGRP-targeting monoclonal antibodies, galcanezumab [19] and fremanezumab [20], indicate no significant development or worsening hypertension when compared to placebo. As such, it is important to evaluate and understand the cardiovascular safety of all therapies that block the effects of CGRP in patients with migraine, particularly over the long term.

Eptinezumab (eptinezumab-jjmr; ALD403), a humanized immunoglobulin G1 (IgG1) monoclonal antibody that binds the CGRP ligand with high affinity [21], is approved in the United States and other countries for the preventive treatment of migraine in adults. Four clinical trials found eptinezumab administered via 30-min intravenous infusion every 12 weeks to be safe, well tolerated, and effective in the prevention of episodic and chronic migraine [22–25]. These trials evaluated a range of eptinezumab doses, including 10 mg, 30 mg, 100 mg, 300 mg, and 1000 mg, and included a phase 1b trial in patients with episodic migraine (EM), a phase 2 trial in patients with chronic migraine (CM), and the two pivotal phase 3 trials, PROMISE-1 in patients with EM and PROMISE-2 in patients with CM. PROMISE-1 and PROMISE-2 found that eptinezumab 100 mg and 300 mg met the primary efficacy endpoint of significantly reducing mean monthly migraine headache days over weeks 1–12 [24, 25], with preventive benefits observed in the 24 h after dosing [26]. Based on these findings, the approved doses in the United States are eptinezumab 100 mg and 300 mg administered intravenously for the prevention of migraine. In addition, the follow-up PREVAIL trial found that improvements in patient-reported outcomes were generally sustained through week 104 [27].

To determine the effect of eptinezumab on cardiovascular co-morbidities and events in patients with migraine, the present analysis pooled the patient-level safety data from the placebo-controlled clinical trial program for eptinezumab in migraine prevention, focusing specifically on the approved 100-mg and 300-mg eptinezumab doses. This analysis also included the unapproved high dose of 1000 mg, which is over 3 times the maximum approved dose for eptinezumab. To evaluate the effect of eptinezumab treatment on cardiovascular co-morbidities and events in patients with EM or CM, this analysis determined if the rate of cardiovascular adverse events (AEs) was different for patients treated with eptinezumab, compared with patients receiving placebo. A secondary analysis was conducted to investigate the effects of treatment on blood pressure.

## Methods

### Design

Detailed methodology for four trials in the eptinezumab clinical trial program have been published previously [22–25]. In addition, the integrated safety analysis of all human studies involved in the clinical development has also been published [28]. The studies included in this pooled analysis are detailed in Table 1. The PREVAIL study, which was an open-label study in which patients were treated with eptinezumab 300 mg for up to 2 years, was not included in this integrated analysis due to the lack of a placebo arm [27].

**Table 1:** Eptinezumab Clinical Studies

Study	Description	Migraine Diagnosis	Dosing Frequency	Follow-up Visits
NCT01772524 [22]	Phase 1b DB/PC/PG/R	EM	1000 mg, placebo Single dose (day 0)	Weeks 4, 8, 12, 24
NCT02275117 [23]	Phase 2 DB/PC/PG/R	CM	10, 30, 100, 300 mg, placebo Single dose (day 0)	Weeks 4, 8, 12, 24, 36, 49
PROMISE-1 (NCT02559895) [24]	Phase 3 DB/PC/PG/R	EM	30, 100, 300 mg, placebo Four doses (day 0, weeks 12, 24, 36)	Weeks 4, 8, 12, 16, 20, 24, 28, 36, 48, 56
PROMISE-2 (NCT02974153) [25]	Phase 3 DB/PC/PG/R	CM	100, 300 mg, placebo Two doses (day 0, week 12)	Weeks 2, 4, 8, 12, 16, 20, 24, 32

*DB* Double-blind, *PC* Placebo-controlled, *PG* Parallel-group, *R* Randomized

### Reporting

Study protocols and safety reporting documents for individual trials were reviewed in each of the regions where the studies were conducted, with each phase 3 trial (PROMISE-1, PROMISE-2) having an active Data and Safety Monitoring Board. Study documents, including data collection methods, were reviewed and approved at a local investigator site level before study initiation, with each site approved for appropriateness and ability to complete the required documentation. During study conduct, all information related to AEs was either spontaneously reported by the patient or in response to the investigator’s non-directed questioning regarding safety, per the standard procedures recommended by the FDA and outlined in the clinical study protocols. For the purposes of this analysis, any reported AEs and all laboratory data (including blood pressure results) for the four placebo-controlled migraine prevention studies were then integrated and pooled.

### Eligibility criteria

All four studies had similar inclusion/exclusion criteria, with each study having a screening period of 4 weeks, followed by a 4-week baseline period to confirm study eligibility before the patient was randomized. Key inclusion criteria at screening were age 18 years or older and a history of migraine with or without aura for at least 12 months. The CM studies included patients who experienced at least 15 headache days per month during the baseline period, of which at least 8 were documented migraine days. The EM studies included patients who reported between 4 and 14 headache days per month during the baseline period, of which at least 4 were reported as migraine days.

In the phase 3, PROMISE-1 EM study, the use of medications for acute headache before screening was restricted to acute migraine-specific medications (triptan or ergots; limited to ≤10 days per month), analgesics or nonsteroidal anti-inflammatory drugs (NSAIDs; limited to ≤14 days per month), and opioid−/butalbital-containing medications (limited to ≤4 days per month). In the phase 3 PROMISE-2 CM study, there was no restriction on the acute migraine-specific medications, analgesics, or NSAIDs during the screening period, with a total of 431 patients (40.2%) diagnosed with medication-overuse headache at baseline. Patients could not, however, have used opioid- or butalbital-containing medications on >4 days per month during the 3 months prior to enrollment. In the phase 3 migraine prevention studies, after randomization had been completed, patients could use acute migraine-specific medications, analgesics, NSAIDs, or other medications as needed to treat acute migraine.

In the phase 1b, phase 2, and PROMISE-1 studies, patients were excluded due to pre-existing cardiovascular disease (hypertension, ischemic heart diseases), neurological disease, cerebrovascular disease, diabetes, or Raynaud’s disease. In PROMISE-2, patients with active, progressive, or unstable cardiovascular disorders were excluded. In situations that were unclear, the investigator was to contact the medical monitor for guidance. Patients were excluded from the migraine prevention studies if they had a history or evidence of atherosclerosis, cardiomyopathy, coronary artery disease, diabetes, serious heart rhythm abnormalities, or other cardiovascular diseases. The studies for eptinezumab used in this post hoc analysis included patients with allowable cardiovascular risk factors including a medical history of hyperlipidemia-related conditions or conditions leading to higher likelihood of diabetes development; obesity (Class I, Class II, and Class III); an age range of 18 to 75 years (including male patients aged ≥45 years, and female patients aged ≥55 years); and race (including patients who reported as Black or African American) [29]. Patients with a medical history of hyperlipidemia-related conditions included dyslipidemia, hypercholesterolemia, hyperlipidemia, hypertriglyceridemia, or lipid metabolism disorder; conditions leading to higher likelihood of diabetes development included glucose tolerance impaired, hyperglycemia, or impaired fasting glucose.

### Outcome measures

In all studies, AEs that emerged after the first dose of study treatment (eptinezumab 100 mg, 300 mg, 1000 mg, or placebo) were recorded through study completion. The Medical Dictionary for Regulatory Activities (MedDRA) was used in all of the studies (Version 15.0 in phase 1 and 2 trials and version 20.1 in phase 3 trials) to determine the preferred term and system organ class for each AE, with severity graded according to Common Terminology Criteria for Adverse Events (CTCAE) criteria, version 4.03. Per the CTCAE criteria, a serious AE was defined as having met at least 1 of the following criteria: fatal, life-threatening, requiring inpatient hospitalization or prolongation of existing hospitalization, resulting in persistent or significant disability/incapacity, congenital anomaly/birth defect, or another medically important event. During the phase 3 clinical trials, a Data Monitoring Committee adjudicated each potential cardiovascular treatment-emergent adverse event (TEAE) based on pre-specified definitions, including death, acute myocardial infarction or hospitalization for unstable angina event, non-fatal stroke or transient ischemic attack, coronary revascularization procedure, hospitalization for hypertension, hospitalization for peripheral artery disease, or revascularization procedure for peripheral artery disease. Study drug discontinuation and infusion interruption due to cardiovascular TEAEs was monitored, as were any new or changed cardiovascular concomitant medications.

In all studies, vital signs measurements, including resting blood pressure and heart rate, were captured at the baseline visit, each 12-week study visit for dosing, and the study conclusion visit. In PROMISE-2, measurements were captured prior to dosing and at 2 h (±30 min) after administration end; in all other studies, measurements were captured prior to dosing and at 4 h (±30 min) after administration end.

### Statistical analysis

Integrated analyses of AEs were conducted by assigned treatment (eptinezumab 100 mg, 300 mg, 1000 mg, or placebo), with patients summarized within the treatment group that they received. For patients treated with two different dose levels, they were summarized in the treatment arm of the highest dose level received. No patient who received eptinezumab 1000 mg received any other dose level because the phase 1 (NCT01772524) was single-dose and because patients in the phase 1 and phase 2 (NCT02275117) studies were excluded from the phase 3 (NCT02559895 or NCT02974153) studies. To analyze for specific cardiovascular AEs, standardized MedDRA queries with narrow search terms were used to determine the overall incidences in each treatment group in the following categories: ischemic central nervous system vascular conditions, ischemic heart disease, peripheral artery disease, and hypertension. The integrated pooled population was utilized to summarize resting systolic blood pressure (BP), diastolic BP, and pulse rate at each study visit, with mean values (± standard deviation [SD]) calculated.

## Results

The main results of the four trials have been published previously [22–25] and, in general, eptinezumab was found to be safe, well-tolerated, and efficacious in preventing migraine. Demographics and baseline characteristics for the individual clinical trials have been reported [22–25] and were similar among treatment and placebo groups. Baseline demographics and characteristics of the pooled population are presented in Table 2, with the groups being well matched across baseline characteristics. A total of *N* = 2268 patients with migraine were included in this analysis, including patients receiving eptinezumab 100 mg, *n* = 701; eptinezumab 300 mg, *n* = 695; eptinezumab 1000 mg, *n* = 81; placebo, *n* = 791. Regarding cardiovascular risk factors, 31.3% of patients were obese (Class I or II), and about half of the population was ≥40 years. Approximately 48% and 13% of the patient population had ≥1 and ≥2 cardiovascular risk factors, respectively. Although the individual trials varied in number of doses administered and overall treatment duration, more than 97% of patients had study drug exposure for ≥12 weeks (see Table 3).

**Table 2:** Baseline Demographics and Characteristics

	Eptinezumab 100 mg	Eptinezumab 300 mg	Eptinezumab 1000 mg	Placebo
	*N* = 701	*N* = 695	*N* = 81	*N* = 791
Mean age, years (SD)	39.9 (11.11)	40.1 (10.84)	38.6 (10.81)	39.3 (10.96)
Sex: Female, n (%)	590 (84.2)	611 (87.9)	67 (82.7)	686 (86.7)
Race, n (%)				
White	636 (90.7)	623 (89.6)	66 (81.5)	677 (85.6)
Black / African American	50 (7.1)	54 (7.8)	10 (12.3)	84 (10.6)
Asian	2 (0.3)	3 (0.4)	4 (4.9)	7 (0.9)
American Indian / Alaska Native	1 (0.1)	3 (0.4)	0	3 (0.4)
Native Hawaiian / other Pacific Islander	1 (0.1)	2 (0.3)	0	2 (0.3)
Multiple races	9 (1.3)	8 (1.2)	1 (1.2)	13 (1.6)
Other	2 (0.3)	1 (0.1)	0	5 (0.6)
Not reported	0	1 (0.1)	0	0
Mean BMI, kg/m^2^	27.6 (6.15)	27.3 (5.91)	27.5 (5.17)	27.9 (6.14)
Preventive migraine medication use, n (%)*	52/478 (10.9)	62/471 (13.2)	–	49/487 (10.1)
Cardiovascular risk factors, n (%)				
Hypertension-related	36 (5.1)	26 (3.7)	1 (1.2)	28 (3.5)
Hyperlipidemia-related	48 (6.8)	45 (6.5)	5 (6.2)	45 (5.7)
Diabetes-related	3 (0.4)	0	1 (1.2)	5 (0.6)
History of ischemic CV events/procedures	4 (0.6)	3 (0.4)	0	3 (0.4)
Obesity (Class 1 or 2), BMI ≥30 kg/m^2^	226 (32.2)	197 (28.3)	26 (32.1)	265 (33.5)
Male ≥45 years	43 (6.1)	24 (3.5)	4 (4.9)	38 (4.8)
Female ≥55 years	63 (9.0)	57 (8.2)	2 (2.5)	49 (6.2)
Black or African American race	50 (7.1)	54 (7.8)	10 (12.3)	84 (10.6)
≥ 1 CV risk factor	350 (49.9)	316 (45.5)	41 (50.6)	382 (48.3)
≥ 2 CV risk factors	97 (13.8)	83 (11.9)	7 (8.6)	112 (14.2)
Medical/Surgical CV history, n (%)				
Angina pectoris	0	1 (0.1)	0	0
Aortic valve incompetence	1 (0.1)	0	0	0
Arrhythmia	0	0	1 (1.2)	1 (0.1)
Supraventricular arrhythmia	0	1 (0.1)	0	0
Atrioventricular block, first degree	0	0	0	1 (0.1)
Bradycardia	2 (0.3)	4 (0.6)	0	4 (0.5)
Bundle branch block, left	1 (0.1)	0	0	0
Bundle branch block, right	0	2 (0.3)	0	2 (0.3)
Cardiomegaly	1 (0.1)	0	0	0
Cardiomyopathy	0	1 (0.1)	0	0
Mitral valve incompetence	1 (0.1)	0	0	1 (0.1)
Mitral valve prolapse	1 (0.1)	4 (0.6)	0	6 (0.8)
Palpitations	3 (0.4)	2 (0.3)	0	5 (0.6)
POTS	1 (0.1)	0	0	0
Sinus arrhythmia	1 (0.1)	1 (0.1)	0	0
Sinus bradycardia	0	0	0	2 (0.3)
Sinus tachycardia	1 (0.1)	1 (0.1)	0	1 (0.1)
Supraventricular tachycardia	1 (0.1)	2 (0.3)	0	1 (0.1)
Tachycardia	0	2 (0.3)	1 (1.2)	5 (0.6)
Tricuspid valve incompetence	1 (0.1)	0	0	0
Ventricular extrasystoles	1 (0.1)	0	0	1 (0.1)
Ventricular tachycardia	0	0	0	1 (0.1)

*Use of a stable preventive migraine medication regimen was permitted in CM studies, and as such, the number of patients reflects only those studies. *BMI* body mass index, *CM* Chronic migraine, *CV* Cardiovascular, *POTS* Postural orthostatic tachycardia syndrome, *SD* Standard deviation

**Table 3:** Clinical Study Duration

	Eptinezumab 100 mg	Eptinezumab 300 mg	Eptinezumab 1000 mg	Placebo
	*N* = 701	*N* = 695	*N* = 81	*N* = 791
Total Time in Clinical Study, n (%)				
≥12 weeks	680 (97.0)	681 (98.0)	78 (96.3)	766 (96.8)
≥24 weeks	642 (91.6)	649 (93.4)	46 (56.8)	692 (87.5)
≥36 weeks	288 (41.1)	293 (42.2)	N/A	280 (35.4)
≥48 weeks	263 (37.5)	270 (38.8)	N/A	256 (32.4)

Total Exposure Time = Last day on study – first dose date + 1. *N/A* Not applicable

### Cardiovascular treatment-emergent adverse events

The pooled patient-level analysis of cardiovascular TEAEs is presented in Table 4. In general, there was a limited incidence of cardiovascular TEAEs across all four clinical trials, with rates similar between the eptinezumab groups and placebo. Cardiovascular TEAEs were mild or moderate in severity (grades 1 and 2, respectively), with none graded 3–5 or as serious or life-threatening AEs. The evaluation of cardiovascular TEAEs stratified by patients with ≥1 and ≥2 cardiovascular risk factors at baseline are presented in Table 5. Although cardiovascular TEAEs were limited and not severe in nature, the majority occurred in patients with ≥1 and ≥2 cardiovascular risk factors at baseline and no differences in incidence were reported between the eptinezumab- and placebo-treated patient groups. Also, there was no signal of any dose-related incidence in the eptinezumab 100-mg, 300-mg, or 1000-mg treatment arms.

**Table 4:** Treatment-Emergent Cardiovascular Adverse Events

	Eptinezumab 100 mg	Eptinezumab 300 mg	Eptinezumab 1000 mg	Placebo
*Patients, n (%)*	*N* = 701	*N* = 695	*N* = 81	*N* = 791
Cardiac disorders	6 (0.9)	8 (1. 2)	2 (2.5)	8 (1.0)
Bradycardia	0	2 (0.3)	0	0
Palpitations	1 (0.1)	2 (0.3)	0	3 (0.4)
Tachycardia	1 (0.1)	2 (0.3)	0	1 (0.1)
Atrial fibrillation	0	1 (0.1)	0	1 (0.1)
Atrioventricular block first degree	2 (0.3)	1 (0.1)	0	1 (0.1)
Bundle branch block right	0	0	1 (1.2)	1 (0.1)
Nodal rhythm	0	0	1 (1.2)	0
Sinus bradycardia	0	0	0	1 (0.1)
Sinus tachycardia	1 (0.1)	0	0	0
Supraventricular extrasystoles	1 (0.1)	0	0	0
Investigations^a^	37 (5.3)	28 (4.0)	10 (12.3)	39 (4.9)
Increased blood pressure	7 (1.0)	3 (0.4)	1 (1.2)	5 (0.6)
Increased heart rate	0	1 (0.1)	0	0
Increased systolic blood pressure	1 (0.1)	0	0	0
Abnormal electrocardiogram Q wave	1 (0.1)	0	1 (1.2)	0
Prolonged electrocardiogram QT	0	0	3 (3.7)	1 (0.1)
Vascular disorders	11 (1.6)	10 (1.4)	1 (1.2)	8 (1.0)
Hot flush	2 (0.3)	4 (0.6)	1 (1.2)	0
Hypertension	4 (0.6)	3 (0.4)	0	6 (0.8)
Flushing	1 (0.1)	2 (0.3)	0	1 (0.1)
Hypotension	1 (0.1)	1 (0.1)	0	0
Prehypertension	0	0	0	1 (0.1)

^a^Not limited to CV-related investigations. *CV* Cardiovascular

**Table 5:** Treatment-Emergent Cardiovascular Adverse Events in Patients with Cardiovascular Risk Factors at Baseline

	Eptinezumab 100 mg	Eptinezumab 300 mg	Eptinezumab 1000 mg	Placebo
*Patients with at least 1 cardiovascular risk factor, n (%)*	*N* = 350	*N* = 316	*N* = 41	*N* = 382
Any TEAE	196 (56.0)	187 (59.2)	22 (53.7)	220 (57.6)
Cardiac or Vascular TEAEs	13 (3.7)	14 (4.4)	4 (9.8)	19 (5.0)
Increased blood pressure	5 (1.4)	3 (0.9)	0	2 (0.5)
Hypertension	2 (0.6)	1 (0.3)	0	5 (1.3)
Hot flush	0	3 (0.9)	0	0
Palpitations	1 (0.3)	1 (0.3)	0	3 (0.8)
Syncope	2 (0.6)	2 (0.6)	0	3 (0.8)
Tachycardia	0	2 (0.6)	0	1 (0.3)
Chest pain	0	0	1 (2.4)	1 (0.3)
ECG QT prolongation	0	0	2 (4.9)	1 (0.3)
Flushing	0	1 (0.3)	0	1 (0.3)
Sinus tachycardia	1 (0.3)	0	0	0
Increased systolic blood pressure	1 (0.3)	0	0	0
Abnormal ECG Q wave	0	0	1 (2.4)	0
Increased heart rate	0	1 (0.3)	0	0
Hypotension	1 (0.3)	0	0	0
Atrial fibrillation	0	0	0	1 (0.3)
Prehypertension	0	0	0	1 (0.3)
**Patients with at least 2 cardiovascular risk factors, n (%)**	*N* = 97	*N* = 83	*N* = 7	*N* = 112
Any TEAE	54 (55.7)	51 (61.4)	4 (57.1)	69 (61.6)
Cardiac or Vascular TEAEs	8 (8.2)	4 (4.8)	0	8 (7.1)
Increased blood pressure	2 (2.1)	1 (1.2)	0	1 (0.9)
Hypertension	2 (2.1)	1 (1.2)	0	4 (3.6)
Syncope	2 (2.1)	1 (1.2)	0	2 (1.8)
Palpitations	1 (1.0)	0	0	0
Hot flush	0	1 (1.2)	0	0
Sinus tachycardia	1 (1.0)	0	0	0
Atrial fibrillation	0	0	0	1 (0.9)

*ECG* Electrocardiogram, *TEAE* Treatment-emergent adverse event

In total, 27 cardiac or vascular TEAEs occurred that were deemed directly related to treatment. Of these, 8 occurred in patients treated with eptinezumab 100 mg (*n* = 1 each for sinus tachycardia, increased BP, increased systolic BP, ECG ST segment depression, hypertension, and flushing; *n* = 2 for hot flushes), 7 occurred in patients treated with eptinezumab 300 mg (*n* = 1 each for atrioventricular first degree block, ECG T-wave inversion, increased heart rate; *n* = 2 each for hot flushes and flushing), 6 occurred in patients treated with eptinezumab 1000 mg (*n* = 1 each for ECG abnormal Q-wave, ECG abnormal T-wave, and hot flush; *n* = 3 for ECG QT prolongation), and 6 occurred in patients treated with placebo (n = 1 each for ECG abnormal T-wave, hypertension, flushing, and pre-hypertension; *n* = 2 for palpitations).

The incidence of study drug discontinuations and infusion interruptions due to TEAEs are available in Supplemental Table 1. Across the pooled population comprising the four eptinezumab migraine prevention studies, a total of 9 (1.3%) and 13 (1.9%) patients in the eptinezumab 100-mg and eptinezumab 300-mg groups, respectively, and 8 (1.0%) patients receiving placebo discontinued study drug due to TEAEs; no patient discontinued in the eptinezumab 1000-mg group due to TEAEs. Hypertension led to 1 (0.1%) patient receiving 100 mg eptinezumab discontinuing study drug use. However, no patients discontinued in the 300- or 1000-mg eptinezumab groups or in the placebo groups, and no other cardiovascular TEAEs resulted in treatment discontinuation. A total of 11 (1.6%), 13 (1.9%), and 6 (0.8%) patients in the eptinezumab 100-mg, eptinezumab 300-mg, and placebo groups, respectively, had an infusion interruption due to TEAEs; none were cardiovascular in nature. No patients had infusion interruptions due to a TEAE in the eptinezumab 1000-mg group.

**Fig. 1:**
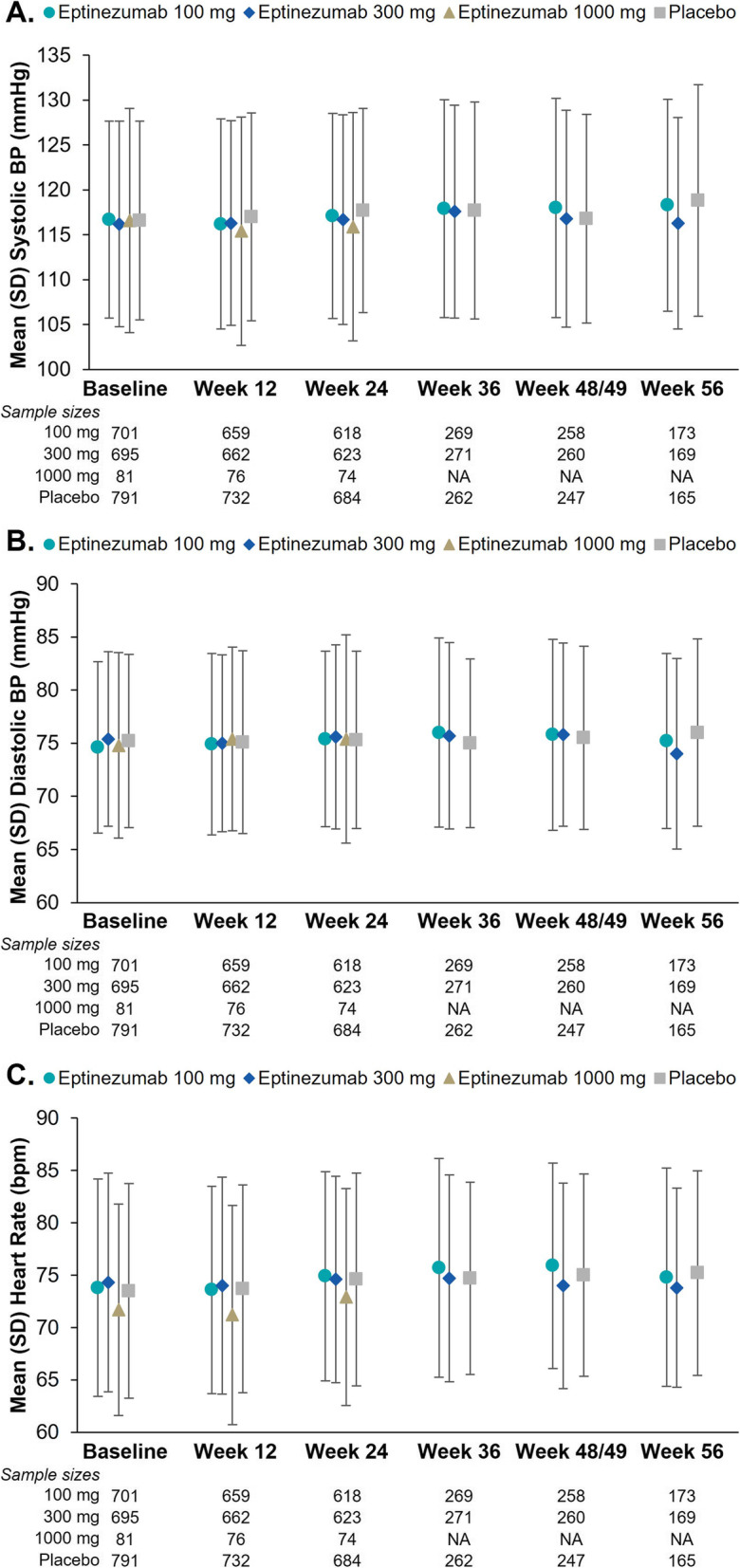
Mean Vital Sign Parameters Over Time: (A) Systolic Blood Pressure (BP), (B) Diastolic BP, and (C) Heart Rate

The assessment of vital signs over time in the pooled population is presented in Fig. 1, with the readings being similar across the treatment groups and across the study visits. Mean (SD) change from baseline to week 12 in systolic BP (see Fig. 1A) was −0.4 (10.2) mm Hg in the eptinezumab 100-mg group, 0.2 (10.6) mm Hg in the eptinezumab 300-mg group, −1.0 (11.8) mm Hg in the eptinezumab 1000-mg group, and 0.5 (10.5) mm Hg in the placebo group. Mean (SD) change from baseline to week 12 in diastolic BP (see Fig. 1B**)** was 0.4 (8.2) mm Hg in the eptinezumab 100-mg group, −0.3 (7.9) mm Hg in the eptinezumab 300-mg group, 0.7 (7.9) mm Hg in the eptinezumab 1000-mg group, and −0.1 (7.5) mm Hg in the placebo group. Similar results were observed at week 24 (100 mg, 300 mg, 1000 mg, and placebo) as well as through week 56 (100 mg, 300 mg, and placebo only). There were no clinically relevant differences across treatment groups. Similar results were observed in changes in resting heart rate (see Fig. 1C), with a mean (SD) change from baseline to week 12 of 0.0 (9.7) beats per minute (bpm) in the eptinezumab 100-mg group, −0.2 (10.4) bpm in the eptinezumab 300-mg group, −0.5 (9.8) bpm in the eptinezumab 1000-mg group, and 0.4 (9.8) bpm in the placebo group. Similar results were observed at week 24 and week 56, with no clinically relevant differences across treatment groups.

The analysis of new or changed concomitant cardiovascular medications is presented in Supplemental Table 2. In general, treatment with eptinezumab (any dose) did not lead to new or changed cardiovascular-related concomitant medications, with the overall rates similar to placebo.

## Discussion

Across the four clinical studies, the incidences of AEs were similar across eptinezumab and placebo treatment groups, with no evidence of dose dependency for eptinezumab with regard to cardiovascular safety outcomes. The review of both patient-level and aggregate AEs did not find evidence of an association between eptinezumab treatment and any cardiovascular event. Based on the low number of cardiovascular events that occurred in any treatment group, it was not possible to conduct any further subgroup analysis, such as in patients with acute migraine-specific medication use at baseline. There was no evidence that patients treated with eptinezumab who used acute migraine-specific medications had any greater incidence of serious adverse events compared with non-users of acute migraine-specific medications. Because CGRP contributes to the maintenance and regulation of cardiovascular homeostasis, these results are of clinical relevance for patients with migraine and health care providers [30].

Among patients with at least one cardiovascular risk factor at baseline, the incidence of AEs was similar across the eptinezumab and placebo treatment groups and independent of the presence (or absence) of cardiovascular risk factors at baseline. Similarly, eptinezumab treatment for up to 56 weeks had no relevant effect on blood pressure or heart rate, with no differences across treatment groups, including in patients who received eptinezumab 1000 mg. Collectively, these pooled, patient-level analyses support the cardiovascular safety profile of eptinezumab in the prevention of migraine across different study periods (up to 56 weeks), patient populations (EM and CM), and methodologies (single and multiple IV infusions), providing a comprehensive evaluation of the overall safety of eptinezumab for prevention of migraine.

### Limitations

This analysis of cardiovascular safety utilized patient-level data that were derived as part of the controlled setting of clinical trials. To generalize these data, additional evidence from real-world analyses would be needed to confirm the cardiovascular safety of eptinezumab in all patient populations. In addition, cardiovascular safety was not analyzed specifically in patients with migraine with aura, a known associate of cardiovascular comorbidities; however, a separate analysis found no increased risk for cardiovascular TEAEs in these patients [31]. Further, the studies used in this analysis excluded patients with evidence of significant cardiovascular disease (including those with uncontrolled or newly diagnosed hypertension) and diabetes; therefore, the long-term safety in high-risk patients remains unknown. Another limitation to this analysis is that tobacco use was not documented. While triptans and ergots have been associated with a higher risk of cardiovascular TEAEs [17, 32, 33], we did not examine specific use of these acute migraine-specific medications in our patients due to small subgroup sizes.

### Conclusions

This pooled, patient-level analysis of four clinical trials in patients with EM or CM found that eptinezumab at doses of 100 mg, 300 mg, and 1000 mg (which is more than 3 times the highest maximum approved dose) did not result in meaningful changes in blood pressure, heart rate, or concomitant cardiovascular-medication usage, and had comparable incidence of cardiovascular TEAEs to placebo. The overall incidence of cardiovascular TEAEs was low.

## Supplementary Information

**Additional file 1: Supplemental Table 1.** Study Drug Discontinuation and Infusion Interruption Due to Cardiovascular Treatment-emergent Adverse Events Across the Pooled Eptinezumab Clinical Trial Population. **Supplemental Table 2.** New or Changed Cardiovascular Concomitant Medications Across the Pooled Eptinezumab Clinical Trial Population.

## Data Availability

In accordance with EFPIA’s and PhRMA’s “Principles for Responsible Clinical Trial Data Sharing” guidelines, Lundbeck is committed to responsible sharing of clinical trial data in a manner that is consistent with safeguarding the privacy of patients, respecting the integrity of national regulatory systems, and protecting the intellectual property of the sponsor. The protection of intellectual property ensures continued research and innovation in the pharmaceutical industry. Deidentified data are available to those whose request has been reviewed and approved through an application submitted to https://www.lundbeck.com/trials.

## Abbreviations

AE: Adverse event
BP: Blood pressure
CV: Cardiovascular
CGRP: Calcitonin gene-related peptide
CM: Chronic migraine
CTCAE: Common terminology criteria for adverse events
EM: Episodic migraine
IgG1: Immunoglobulin G1
MedDRA: Medical Dictionary for Regulatory Activities
NSAID: Nonsteroidal anti-inflammatory drug
SD: Standard deviation
TEAE: Treatment-emergent adverse event
