# Supplementary material for: Cardiovascular outcomes in adults with migraine treated with eptinezumab for migraine prevention: pooled data from four randomized, double-blind, placebo-controlled studies
Source: J Headache Pain. 2021 Nov 25;22(1):143. doi: 10.1186/s10194-021-01360-1 (PMC8903619; doi:10.1186/s10194-021-01360-1)
Supplement: Supplementary file 1 — Additional file 1: Supplemental Table 1. Study Drug Discontinuation and Infusion Interruption Due to Cardiovascular Treatment-emergent Adverse Events Across the Pooled Eptinezumab Clinical Trial Population. Supplemental Table 2. New or Changed Cardiovascular Concomitant Medications Across the Pooled Eptinezumab Clinical Trial Population. [file 10194_2021_1360_MOESM1_ESM.pdf]

TITLE PAGE

Cardiovascular Outcomes in Adults With Migraine Treated With Eptinezumab for Migraine Prevention: Pooled Data From Four Randomized, Double-Blind, Placebo-Controlled Studies

Timothy R. Smith,[1] Egilius L.H. Spierings,[2] Roger Cady,[3] Joe Hirman,[4] Anders Ettrup,[5] Vivienne Shen[6]

[1]StudyMetrix Research, Saint Peters, MO, USA; [2]Medvadis Research Corporation, Boston PainCare, Waltham, MA, USA; [3]Lundbeck La Jolla Research Center, San Diego, CA, USA; [4]Pacific Northwest Statistical Consulting, Inc., Woodinville, WA, USA; [5]H. Lundbeck A/S, Copenhagen, Denmark; [6]Lundbeck LLC, Deerfield, IL, USA

Corresponding Author:

Timothy R. Smith, MD, RPh

StudyMetrix Research, LLC

3862 Mexico Road

St. Peters, MO 63303

Tel: 636.387.5100

Email: tsmith@studymetrix.com

Supplemental Table 1. Study Drug Discontinuation and Infusion Interruption Due to Cardiovascular Treatment-emergent Adverse Events Across the Pooled Eptinezumab Clinical Trial Population

	Eptinezumab 100 mg	Eptinezumab 300 mg	Eptinezumab 1000 mg	Placebo
	N=701	N=695	N=81	N=791
Study drug discontinuation, n (%)				
**Increased blood pressure**	1 (0.1)	0	0	0
**Hypertension**	1 (0.1)	0	0	0
Infusion interruption, n (%)	0	0	0	0
Possibly related, n (%)	0	0	0	0

Supplemental Table 2. New or Changed Cardiovascular Concomitant Medications Across the Pooled Eptinezumab Clinical Trial Population

	Eptinezumab 100 mg	Eptinezumab 300 mg	Eptinezumab 1000 mg	Placebo
	N=701	N=695	N=81	N=791
Antihypertensives, n (%)*	18 (2.6)	22 (3.2)	5 (6.2)	18 (2.3)
Antilipidemics, n (%)	7 (1.0)	5 (0.7)	2 (2.5)	3 (0.4)
Antianginals, n (%)	0	1 (0.1)	1 (1.2)	1 (0.1)

*Antihypertensives: amlodipine, atenolol, benazepril, betaxolol hydrochloride, candesartan, candesartan cilexetil, captopril, clonidine, clonidine hydrochloride, diltiazem hydrochloride, enalapril, hydrochlorothiazide, hyzaar, lisinopril, losartan, losartan potassium, metoprolol, metoprolol tartrate, nadolol, nicardipine hydrochloride, prazosin, prazosin hydrochloride, propranolol, rilmenidine, spironolactone, timolol, trandolapril, verapamil, verapamil hydrochloride, zestoretic.

**Antilipidemics: atorvastatin, atorvastatin calcium, cholestyramine, fish oil, pravastatin, rosuvastatin, simvastatin.

***Antianginals: glyceryl trinitrate.
